# Attitudinal dataset for mediating the effects of public acceptance on bus reform scheme in a developing country context

**DOI:** 10.1016/j.dib.2019.104035

**Published:** 2019-05-23

**Authors:** Sofyan M. Saleh, Sugiarto Sugiarto, Alfi Salmannur

**Affiliations:** Department of Civil Engineering, Universitas Syiah Kuala, Jl. Tgk. Syech Abdul Rauf No. 7, Banda Aceh 23111, Indonesia

**Keywords:** Stated preference, Public attitude, Bus reform policy, Attitudinal data, CFA, Indonesia

## Abstract

This paper presents a comprehensive dataset of how public perceptions and its statistical implication on the behavioral adaptation toward the acceptability of bus reform scheme proposed by the government of Aceh, Indonesia. Studies indicate that public response to such a proposed policy is significantly related to the effectiveness of its implementation. Given the absence of studies from the developing countries context, a stated preference (SP) questionnaire is developed to investigate the public's consciousness concerning bus reform acceptance. A paper-pencil based questionnaire survey performed by direct interview was conducted in late 2017 and early 2018. The questionnaires were distributed with a total of 450 questionnaires valid. The most pertinent inquiries in our questionnaires were designated to attain (i) attitudinal indicators toward the acceptance of bus reform policy, (ii) attitudinal indicators related to perceived appropriateness of the policy, (iii) perceived awareness of problem private-mode in society, (iv) private-mode dependency, (v) inhibition of freedom of movements and (vi) correct and acceptable policy. We implemented a 4-point Likert scale such as 1 to 4 (strongly disagree to strongly agreed) in order to style the questionnaire easy to answer. A valid dataset was analyzed using Confirmatory Factor Analysis (CFA) for revealing how public perception has statistically significant explained the perceived effectiveness of the proposed bus reform policy.

**Table:** Specifications table

Subject area	Transport Policy, Transport Science
More specific subject area	Travel Behavior Analysis
Type of data	Table and figure
How data was acquired	A paper-pencil based on the Stated Preference survey
Data format	Raw, filtered, analyzed
Experimental factors	A questionnaire distributed among bus ridership and people who live in the surrounding area within the bus lines
Experimental features	The social-demographics, travel attributes and attitudinal questions asked and implemented using a 4-point Likert scale
Data source location	Banda Aceh, Indonesia
Data accessibility	Data are included with this article

**Table:** 

**Value of the data** • Data presents organized dataset of attitudinal data (psychological indicators) in the context of emerging countries related to the travel behavior analysis. • The data provide some essential measurements to offer greater insight related to public consciousness. • The empirical examination could serve as a scale for other studies • Data revealed how public perceptions are statistically significant explained the perceived effectiveness of the proposed bus reform policy by the government.

## 1 Data

**Fig. 1:**
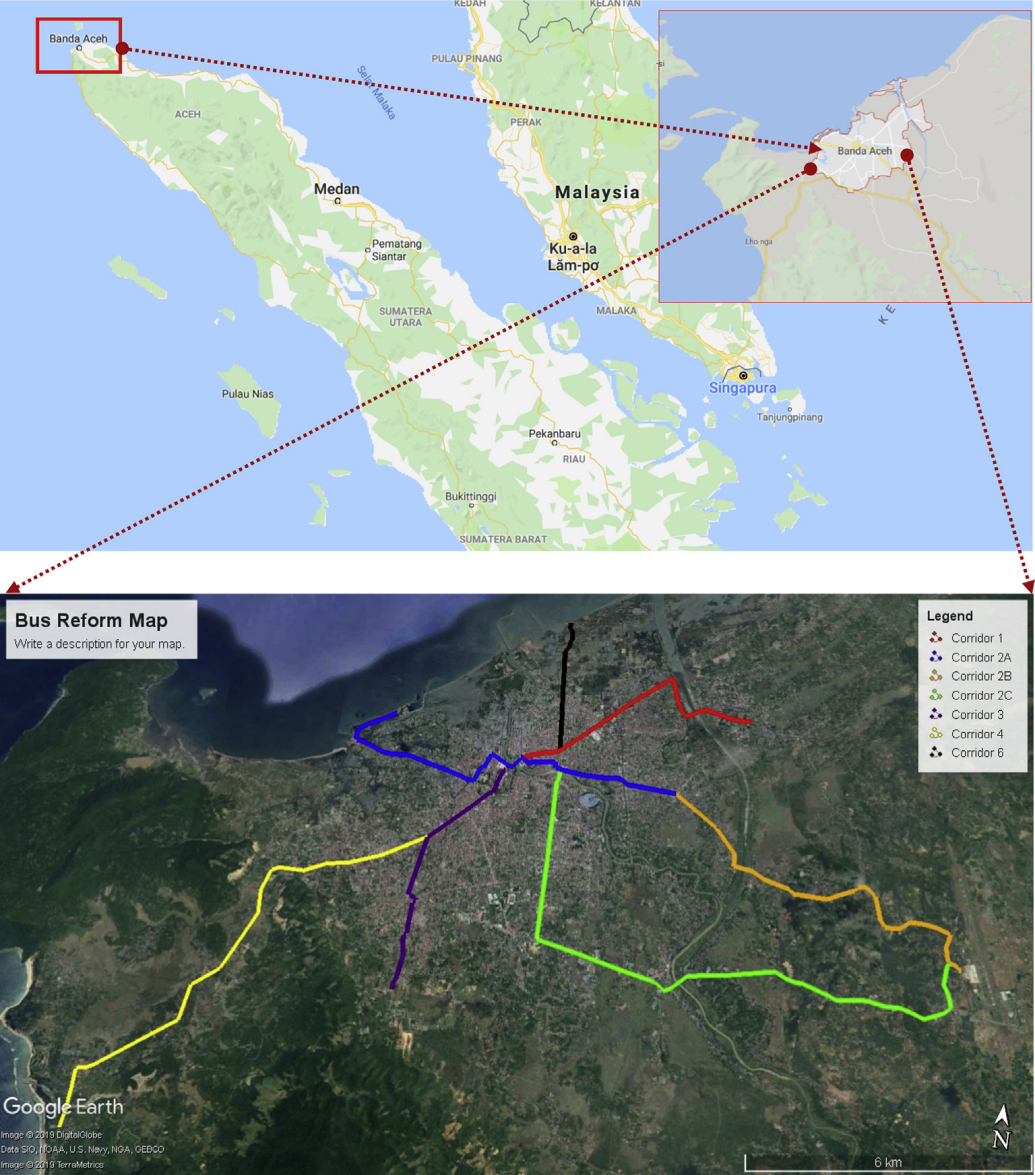
(a) Banda Aceh city, located in the island of Sumatera; (b) Trans Koetaradja corridors (overlay on map data © 2019 Google).

The dataset presents in this paper containing the socio-demographic characteristics, travel attributes, and attitudinal indicators. The dataset obtained from the questionnaire survey using Stated Preference (SP) experiment [1], [2], [3], [4], [5]. Target respondents were commuters, commercial visitors, employees and students within the proposed bus reform policy so-called “Trans Koetaradja” (hereafter “TK”). The TK is a new technology and bus system with a capacity of 60 passengers equipped with an air conditioner, providing more comfort to the passengers [6], [7], [8]. TK reforms required to achieve efficiency and sufficiency of the city bus system. The TK now is under trial runs, a free of charge service is applied within running corridors. The trial run corridors of TK are within the city of Banda Aceh (see Fig. 1a) as the city heavily relies on private modes such as cars and motorcycles [9], [10]. The target area of this study is corridor 1 (red), 3 (dark blue), and 4 (yellow) as shown in Fig. 1b.

The distributions of socio-demographic and travel attributes are described in Table 1 and Table 2. The attitudinal indicators which are consist of the information related to (1) attitudinal indicators toward the acceptance of bus reform policy; (ii) attitudinal indicators related to perceived appropriateness of the policy; (iii) perceived awareness of problem private-mode in society; (iv) private-mode dependency; (v) inhibition of freedom of movements, and (vi) correct and acceptable policy has illustrated in Table 3.

**Table 1:** Distribution of Socio-demographic data.

Item	Category	Number of Samples	Share (%)
Gender	Male	222	49.3%
	Female	228	50.7%
*Total*		*450*	*100%*
Age	19 years or less	95	21.1%
	20–29 years	219	48.7%
	30–39 years	62	13.8%
	40–49 years	32	7.1%
	50–59 years	27	6.0%
	60 years or more	15	3.3%
*Total*		*450*	*100%*
Education	Primary School	300	66.7%
	College	28	6.2%
	University/Bachelor	112	24.9%
	University/post graduate	10	2.2%
*Total*		*450*	*100%*
Monthly Income	1.9 million IDR or less	198	44.0%
	2–3.9 million IDR	157	34.9%
	4–5.9 million IDR	36	8.0%
	6–7.9 million IDR	45	10.0%
	8 million IDR or more	14	3.1%
*Total*		*450*	*100%*
Occupation	Working	122	27.1%
	Student	178	39.6%
	Housewife	66	14.7%
	Unemployed	29	6.4%
	Others	55	12.2%
*Total*		*450*	*100%*
Driver's license	Has driver's license	321	71.3%
	Has no driver's license	129	28.7%
*Total*		*450*	*100%*

**Table 2:** Distribution of mobility/travel attributes.

Item	Category	Number of Samples	Share (%)
Purpose of traveling on the day of the questionnaire survey	Work	95	21.1%
	Meeting and sales	10	2.2%
	Delivery	4	0.9%
	Trader	33	7.3%
	Studying and lessons	168	37.3%
	Entertainment and eating	68	15.1%
	others	72	16.0%
*Total*		*450*	*100%*
Travel mode used on the day of the questionnaire survey	Private mode	387	86.0%
	Public Mode	63	14.0%
*Total*		*450*	*100%*
Frequency of private mode usage in daily life	More than 5 days a week	338	75.1%
	3–4 days a week	40	8.9%
	1–2 days a week	24	5.3%
	1 days a week	15	3.3%
	1 day a month or less	33	7.3%
*Total*		*450*	*100%*
Frequency of public mode usage in daily life	More than 5 days a week	58	12.9%
	3–4 days a week	56	12.4%
	1–2 days a week	36	8.0%
	1 days a week	48	10.7%
	1 day a month or less	252	56.0%
*Total*		*450*	*100%*

**Table 3:** Mediator (latent variable) of attitudinal questions and average score.

Mediator (Latent Variable)	Question ID (Indicator ID)	Content of Attitudinal Questions	Average Score (a 4-point Likert scale 1 to 4
Latent 1: Perceived appropriateness of the policy (PAP)	Q1 (Indicator1)	The bus reform is a correct policy to deal with motorized traffic	3.11
	Q2 (Indicator2)	The bus reform is an acceptable policy	3.16
	Q3 (Indicator3)	The government has given an understanding of the proposed policy	2.74
	Q4 (Indicator4)	The proposed policy is an appropriate policy to reduce congestion	3.10
Latent 2: Private-mode dependency (PDC)	Q5 (Indicator5)	A private-mode is necessary for daily activities	3.22
	Q6 (Indicator6)	Like to drive private-mode (car or motorcycle)	3.00
	Q7 (Indicator7)	Public transport is expensive and has a limited route	2.22
Latent 3: Awareness of problem private-mode in society (APS)	Q8 (Indicator8)	Increasing accident rate in the city	2.94
	Q9 (Indicator9)	Congestion is getting worse in cities	2.82
	Q10 (Indicator10)	Emissions and noise are getting worse in cities	2.85
Latent 4: Inhibition of freedom of movement/less accessibility of the bus (IMA)	Q11 (Indicator11)	The Bus could reduce freedom/maneuver in the road traffic due to the use of large road space	2.37
	Q12 (Indicator12)	The Bus only serves limited routes/access	2.78
	Q13 (Indicator13)	Would be decreased activities if using the Bus	2.56
Latent 5: Correct and acceptable policy (CAP)	Q14 (Indicator14)	The proposed bus reform is correct and acceptable policy	3.10
	Q15 (Indicator15)	The proposed bus reform is a suitable policy to deal with traffic and environmental problem	3.01

## 2 Experimental design and material

The data for our work is consist of socio-economic characteristics, mobility/travel attributes and attitudinal indicators related to public response to the acceptance of Bus Reform policy. As for socio-economic and travel attributes distribution can be seen in Table 1, Table 2.

Concerning with attitudinal data, the SP experimental [11] is applied to deal with attitudinal data in qualitative nature. The SP with a 4-point Likert scale is employed to wide-ranging information related to the attitudinal questions. To gain more assistance by the respondents, the questionnaire form has designed as simple as possible. The vital of our questions are attitudinal intentions toward bus reform policy by the government. In this case, we adopted preceding works done by [11], [12], [13].

**Fig. 2:**
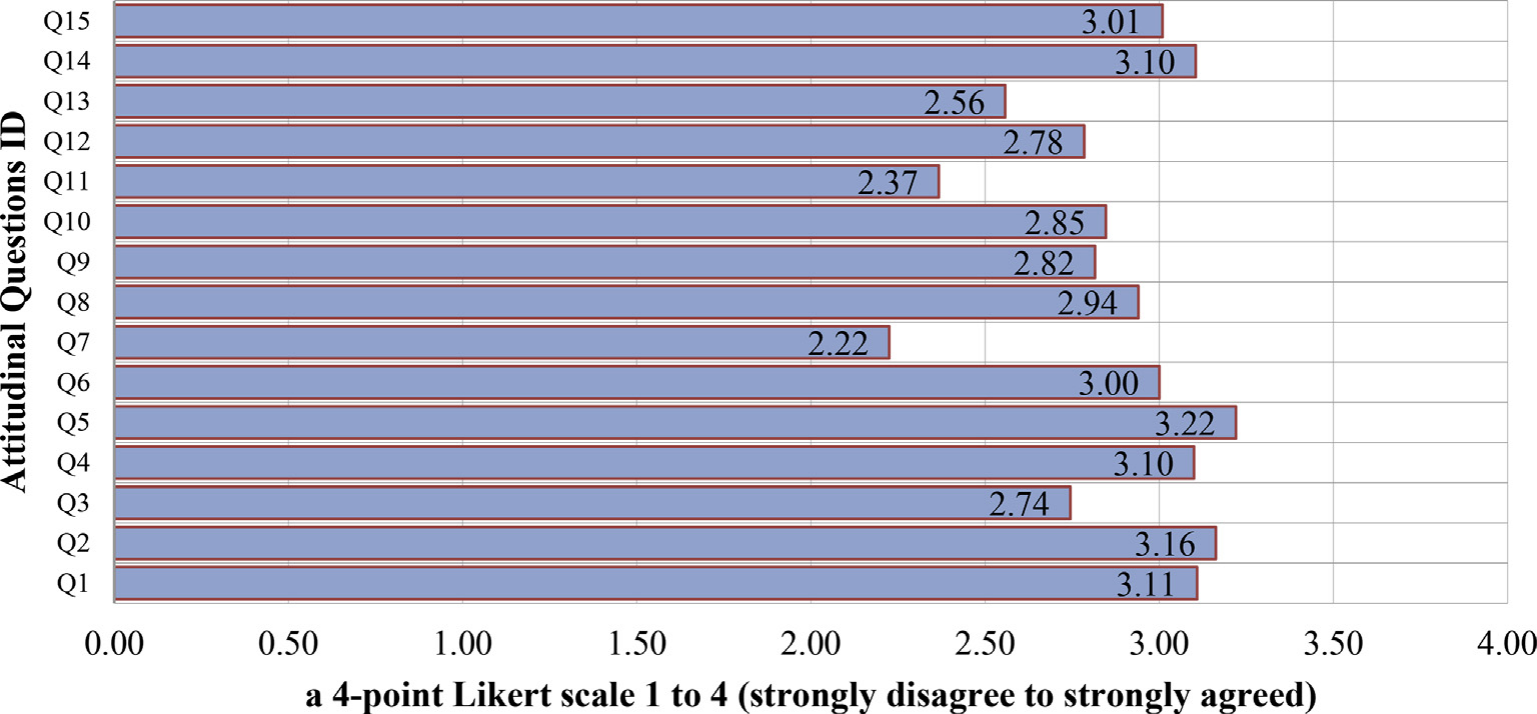
The distribution of the answered responses to attitudinal questions.

Table 3 shows the distribution of the answered responses to attitudinal questions using a 4-point Likert scale 1 to 4 (strongly disagree to strongly agreed) as illustrated in Fig. 2 and summarized in Table 4.

**Table 4:** Summarize of distribution response among a 4-point Likert scale.

Questions ID	A 4-point Likert scale Answered response to attitudinal questions (strongly disagree to strongly agreed)				Total (%)	Average Score
	1 (%)	2 (%)	3 (%)	4 (4%)		
Q1	4.00	8.00	61.11	26.89	100.00	3.11
Q2	1.33	4.22	71.33	23.11	100.00	3.16
Q3	2.00	27.11	65.33	5.56	100.00	2.74
Q4	0.89	8.00	71.33	19.78	100.00	3.10
Q5	1.11	5.78	63.11	30.00	100.00	3.22
Q6	1.56	17.11	61.11	20.22	100.00	3.00
Q7	10.22	60.22	26.44	3.11	100.00	2.22
Q8	1.56	20.67	60.00	17.78	100.00	2.94
Q9	2.22	25.11	61.56	11.11	100.00	2.82
Q10	2.00	24.67	60.00	13.33	100.00	2.85
Q11	5.78	55.33	35.33	3.56	100.00	2.37
Q12	5.11	23.11	60.00	11.78	100.00	2.78
Q13	9.33	34.22	47.78	8.67	100.00	2.56
Q14	2.67	3.78	74.00	19.56	100.00	3.10
Q15	2.22	10.44	71.56	15.78	100.00	3.01

## 3 Statistical framework of analysis

A confirmatory factor analysis (CFA) approach is used to delve more deeply related respondent's psychological intents. The CFA is a kind of structural equation modeling (SEM) that deals with measurement models. That is the relationships between observed measures or indicators such as attitudinal related questions as described in Table 3. Thus, the central concern of CFA is modeling factors or latent variables that are not directly measured but manifest from psychological perceptions or indicators. Therefore, in this study, CFA is applied and used to investigate the determinant factors to accept bus reform policy based on the unobserved variable (latently). Our hypothesized in this work were (1) perceived appropriateness of the policy and awareness of problem private-mode in society would have a positive effect on their intentions to support the proposed policy; (2) determinant of private-mode dependency and inhibition of freedom of movement/less accessibility of the bus would have an adverse consequence on such intentions. By implementing CFA able to delve more deeply related respondent's behavioral intentions respect to the proposed policy. The CFA model as a part of the multiple indicators multiple causes model proposed by [14], [15] systematically the CFA or known as a measurement model, given by:

(1) $$y_{i}=\Lambda \eta_{i}+\zeta_{i}$$

Where y_i_ is a vector of observable attitudinal indicators, ηi is a vector of mediators (latent variables), Λ are matrices of unknown parameters to be calibrated, and the terms ζ_i_ are vector of measurement errors. More application related to the latent variable modeling within a broad of travel behavioral arena can be surveyed in the previous studies done by [1], [2], [3], [4], [5].

**Fig. 3:**
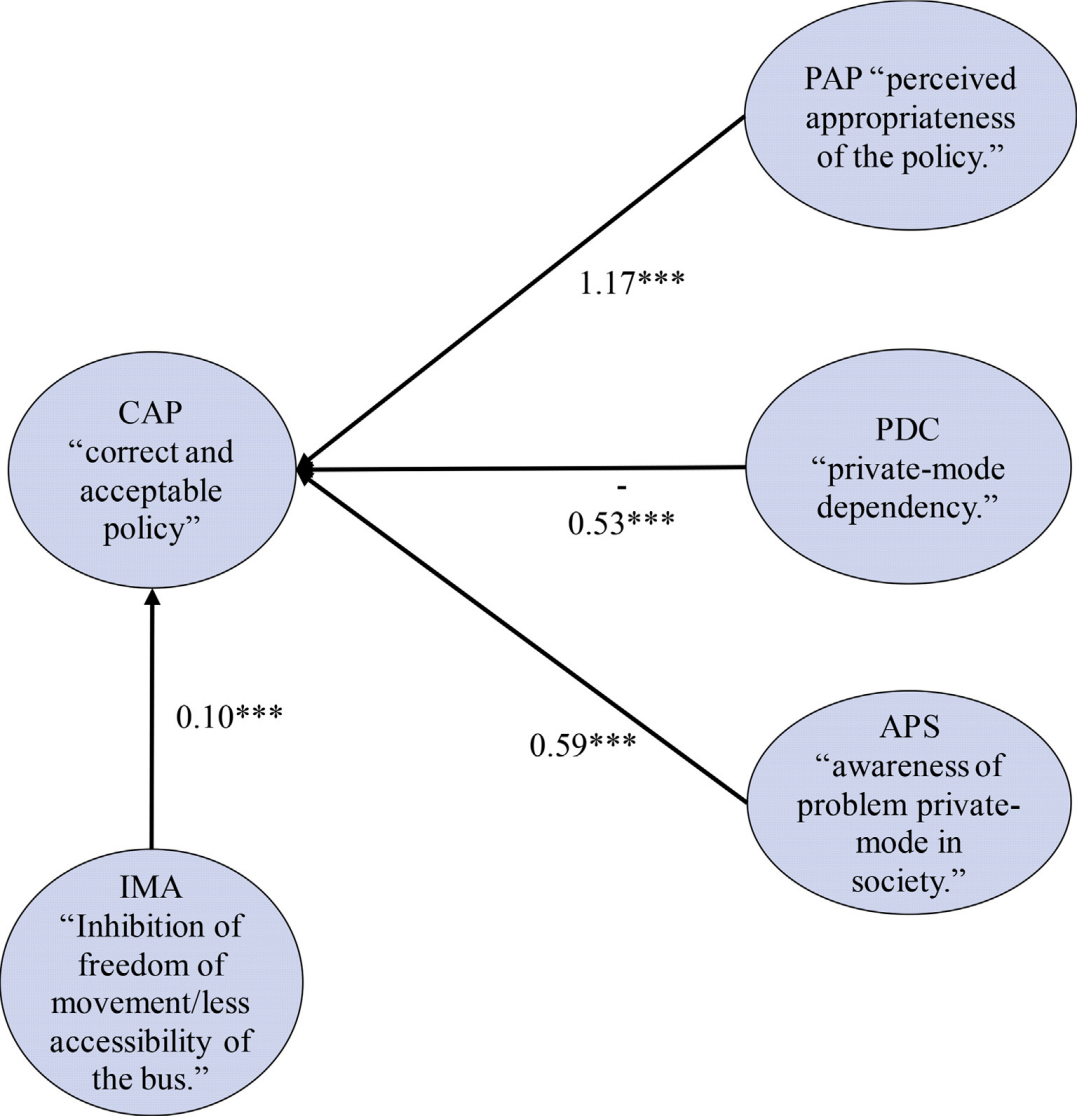
Calibrated loading coefficients among mediators (latent variables). ***path coefficient significant at 1% error level [8].

Statistically speaking, the model and its parameters must be tested using several statistical diagnose tests. We used several model fit indexes to test the goodness of fit model namely the root mean square error of approximation (RMSEA = 0.059), the Adjusted Goodness of Fit Index (AGFI = 0.915), the comparative fit index and the Tucker-Leis index (CFI = 0.939, TFI = 0.972), and χ2/degrees of freedom (χ2/df = 2.58) as described in Table 5. The result of calibrated parameters among mediators (latent 1–5) and its indicators (question Q1-Q15) using CFA can be seen in Table 5. Moreover, the estimated parameters among mediators’ latent variables describe in Fig. 3. For the readers who interested in the discussion about statistical inference from the empirical modeling using CFA are invited to read [8]. Table 6.

**Table 5:** The goodness of Fit (GoF) indices estimated the model.

GoF Indices	Variables	Values	Accepted Thresholds
RMSEA	the root mean square error of approximation	0.059	<0.100
CFI	the comparative fit index	0.939	>0.900
AGFI	the adjusted goodness of fit index	0.915	>0.900
TLI	The Tucker-Lewis index	0.972	>0.900
χ2/df	Chi-square/degrees of freedom	2.580	<3.000

**Table 6:** Calibrated parameters of CFA: coefficients among latent variables (ellipses) and indicators (rectangular); *** path coefficient significant at a 1% error level [8].

Mediator (Latent Variable)	Question ID (Indicator ID)	Content of Attitudinal Questions	Factor Loading/Path Coefficient
Latent 1: Perceived appropriateness of the policy (PAP)	Q1 (Indicator1)	The bus reform is a correct policy to deal with motorized traffic	1.000
	Q2 (Indicator2)	The bus reform is an acceptable policy	0.665***
	Q3 (Indicator3)	The government has given an understanding of the proposed policy	0.555***
	Q4 (Indicator4)	The proposed policy is an appropriate policy to reduce congestion	0.720***
Latent 2: Private-mode dependency (PDC)	Q5 (Indicator5)	A private-mode is necessary for daily activities	1.000
	Q6 (Indicator6)	Like to drive private-mode (car or motorcycle)	−0.790**
	Q7 (Indicator7)	Public transport is expensive and has a limited route	−0.112*
Latent 3: Awareness of problem private-mode in society (APS)	Q8 (Indicator8)	Increasing accident rate in the city	1.000
	Q9 (Indicator9)	Congestion is getting worse in cities	1.208**
	Q10 (Indicator10)	Emissions and noise are getting worse in cities	1.025***
Latent 4: Inhibition of freedom of movement/less accessibility of the bus (IMA)	Q11 (Indicator11)	The Bus could reduce freedom/maneuver in the road traffic due to the use of large road space	1.000
	Q12 (Indicator12)	The Bus only serves limited routes/access	0.269**
	Q13 (Indicator13)	Would be decreased activities if using the Bus	0.681***
Latent 5: A correct and acceptable policy (CAP)	Q14 (Indicator14)	The proposed bus reform is correct and acceptable policy	1.000
	Q15 (Indicator15)	The proposed bus reform is suitable policy to deal with traffic and environmental problem	1.203***

***,**, and *significant at 1%, 5%, and 10% level.

## Appendix The summary of the English translation of the questionnaire

The questionnaire of attitudinal data is structured into five parts of the mediator (latent variable). The attitudinal indicators which are consist of five parts of the information related to (1) attitudinal indicators toward the acceptance of bus reform policy; (ii) attitudinal indicators related to perceived appropriateness of the policy; (iii) perceived awareness of problem private-mode in society; (iv) private-mode dependency; (v) inhibition of freedom of movements, and (vi) correct and acceptable policy. The answered responses to attitudinal questions using a 4-point Likert scale 1 (strongly disagree), scale 2 (disagree), scale 3 (agreed), and scale 4 (strongly agree).

**Part 1:** Perceived appropriateness of the policy (PAP).

Q1 Do you agree that “the bus reform” is a **correct policy** to reduce motorized traffic?
Q2 Do you think that “the bus reform” policy can be **accepted** by the public?
Q3 Do you think that the government has given an **understanding** of the proposed policy?
Q4 Do you think that the proposed policy is an **appropriate policy** to reduce congestion in the city center?

**Part 2:** Private-mode dependency (PDC).

Q5 Do you think a private-mode is **necessary** for daily activities?
Q6 Do you like to **drive private-mode** (car or motorcycle)?
Q7 Do you think that public transport is **expensive and has limited** routes?

**Part 3:** Awareness of problem private-mode in society (APS).

Q8 Do you think that the private-mode (car or motorcycle) traffic could **increase the accident** rate in the city?
Q9 Do you think that **congestion** is getting worse in the city recently?
Q10 Do you think that **emissions and noise** are getting worse in the city recently?

**Part 4:** Inhibition of freedom of movement/less accessibility of the bus (IMA).

Q11 Do you think that the bus could **reduce freedom/maneuver** in the road traffic due to the use of large road space?
Q12 Do you think that the bus only **serves limited routes**/access?
Q13 Do you think that you would be **decreased activities** if using the bus?

**Part 5: Correct and acceptable policy (CAP**).

Q14 Do you think that the proposed bus reform is **correct and acceptable** policy?
Q15 Do you think that the proposed bus reform is a **suitable policy** to deal with traffic and environmental problem?
